# Multi-Function Reflective Vector Light Fields Generated by All-Dielectric Encoding Metasurface

**DOI:** 10.3390/ma15228260

**Published:** 2022-11-21

**Authors:** Qingyu Wang, Chenxia Li, Bo Fang, Xufeng Jing

**Affiliations:** 1 Institute of Optoelectronic Technology, China Jiliang University, Hangzhou 310018, China; 2 Centre for THz Research, China Jiliang University, Hangzhou 310018, China; 3 College of Metrology & Measurement Engineering, China Jiliang University, Hangzhou 310018, China

**Keywords:** metasurface, reflective, vector light

## Abstract

Traditional optics usually studies the uniform polarization state of light. Compared with uniform vector beams, non-uniform vector beams have more polarization information. Most of the research on generating cylindrical vector beams using metasurfaces focuses on generating transmitted beams using the geometric phase. However, the geometric phase requires the incident light to be circularly polarized, which limits the design freedom. Here, an all-dielectric reflective metasurface is designed to generate different output light according to the different polarization states of the incident light. By combining the two encoding arrangements of the dynamic phase and the geometric phase, the output light is a radial vector beam when the linearly polarized light is incident along the x-direction. Under the incidence of linearly polarized light along the y-direction, the generated output light is an azimuthal vector beam. Under the incidence of left-handed circularly polarized light, the generated output light is a vortex beam with a topological charge of −1. Under the incidence of right-handed circularly polarized light, the generated output light is a vortex beam with a topological charge of +1. The proposed reflective metasurface has potential applications in generating vector beams with high integration.

## 1. Introduction

Among the basic properties of light such as intensity, wavelength, phase, and polarization, the research on polarization state with vector properties is the latest development [1,2,3,4,5,6,7,8,9,10]. Traditional optics usually studies the uniform polarization state of light, such as linearly polarized light, circularly polarized light, and elliptically polarized light [11,12,13,14,15,16,17,18,19,20]. In recent years, researchers have paid increasing attention to non-uniformly polarized light [21,22,23,24,25,26,27,28,29,30], such as radially/azimuthally polarized cylindrical vector beams [31,32,33,34,35,36,37,38,39,40,41]. Compared with uniform vector beams, non-uniform vector beams have more polarization information, so their interactions with matter are also more diverse, giving them more potential applications. For example, because the focusing part [42,43] has a larger longitudinal polarization component, tight focusing based on radially-polarized vector light has better characteristics than the traditional focusing system, making it suitable for high-resolution imaging [44], lithography [45], optical trapping [46], and sensing [47].

The traditional methods of generating cylindrical vector beams include birefringence mode selection [48], optical dichroism mode selection [49], photonic crystal mirrors [50], multilayer polarization gratings [51], space-shift phase retarder [52] etc. However, these systems contain various optical devices, so the systems are complex and bulky, and the integration level is low, which hinders the development of vector beams with high integration. With the rise of metasurfaces [53], a new direction has been provided to solve the problem of low integration. Metasurfaces have been widely used in the fields of anomalous refraction [54], lens imaging [55], holographic imaging [56], polarization conversion [57], and the sorting of beams carrying OAM [58,59,60], depending on the content and method of light regulation. On-chip waveguides in integrated photonic devices [61] can also manipulate the polarization state of light through precise deformation control of the waveguide cross-sections, and can be fabricated into on-chip optical vortex detectors [62]. Most of the research on generating cylindrical vector beams using metasurfaces focuses on generating transmission beams by the geometric phase, but the geometric phase requires the incident light to be circularly polarized, which limits the freedom of design.

The terahertz wave is between the visible wave and the microwave. Because the rotational vibration of many biological macromolecules falls in this band, the application of terahertz in biomedicine has great prospects. At the same time, polarized optics have the advantages of carrying a large amount of information without the need for exogenous labels, and are increasingly widely used in biomedicine. The application of cylindrical vector beams in the terahertz band is essential for both biomedicine and terahertz communications.

**Figure 1:**
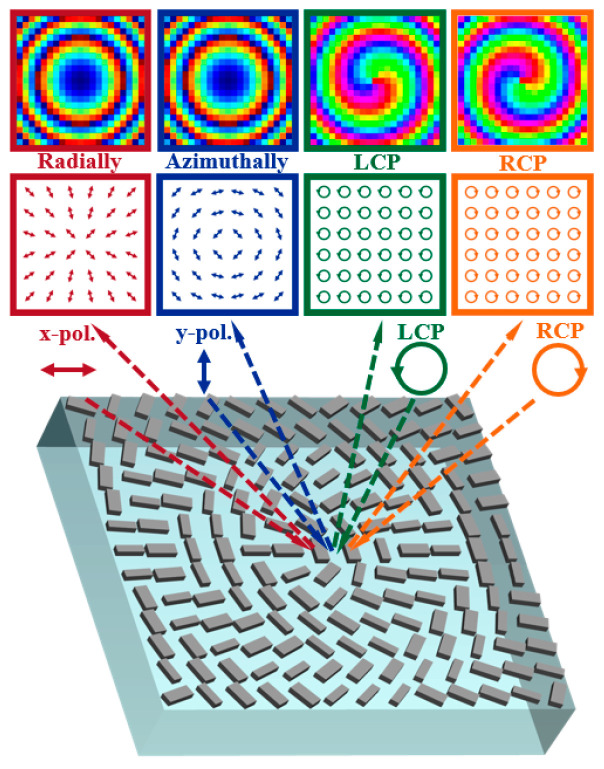
Schematic diagram of the function of reflective metasurfaces.

This paper proposes a method for generating vector beams using a reflective all-dielectric metasurface in the terahertz band through the dynamic phase and the geometric phase. As shown in Figure 1, the different types of beams are generated when the beams with different polarization states are incident on the designed metasurface. Under the incidence of linearly polarized light along the x-direction, the generated light is a radial vector beam. Under the incidence of linearly polarized light along the y-direction, the generated light is an angular vector beam. When a left-handed circularly polarized light is incident, the generated light is a vortex beam with a topological charge of −1. Under the incidence of right-handed circularly polarized light, the generated light is a vortex beam with a topological charge of +1. The proposed method can provide a new approach to designing reflective terahertz micro-nano devices.

## 2. Theory of Multi-Function Metasurface

Considering that the optical characteristics of the metasurface are the same as that of the phase retarder, when the long axis and the short axis of the designed structure coincide with the x-axis and y-axis of the coordinate system, respectively, the transmission matrix can be conveniently simplified and expressed as: $T=\left[\begin{array}{cc} A_{y}e^{i\varphi_{y}} & 0\\ 0 & A_{x}e^{i\varphi_{x}} \end{array}\right]$, where $A_{x}$ and $\varphi_{x}$ are the amplitude and phase of polarized incidence along the x-axis, and $A_{y}$ and $\varphi_{y}$ are the amplitude and phase of polarized incidence along the y-axis. When the structural unit rotates by *θ*, the long and short axes of the structure do not coincide with the x and y axes, and the transfer matrix is expressed as:

(1) $$T^{xy}=R\left(\theta \right)TR\left(-\theta \right)=[\begin{array}{cc} cos\theta & -sin\theta \\ sin\theta & cos\theta \end{array}]\left[\begin{array}{cc} A_{2}e^{i\varphi_{2}} & 0\\ 0 & A_{1}e^{i\varphi_{1}} \end{array}\right]\left[\begin{array}{cc} cos\theta & sin\theta \\ -sin\theta & cos\theta \end{array}\right],$$

Among them, since the long and short axes do not coincide with the x and y axes, $A_{1}$ and $\varphi_{1}$ are the amplitude and phase of the polarized incidence along the x-axis when the structure is not rotated, and $A_{2}$ and $\varphi_{2}$ are the amplitude and phase of the polarized incidence along the y-axis when the structure is not rotated. To improve the polarization conversion efficiency, we can consider the special cases of $A_{1}\approx A_{2}\approx A$ and $\varphi_{1}-\varphi_{2}\approx \pi$. According to Euler’s formula, it can be known that $e^{i\varphi_{1}}=-e^{i\varphi_{2}}$, and at this time, Equation (1) is simplified to:

(2) $$T^{xy}=Ae^{i\varphi_{2}}\left[\begin{array}{cc} cos\theta & -sin\theta \\ sin\theta & cos\theta \end{array}\right]\left[\begin{array}{cc} 1 & 0\\ 0 & -1 \end{array}\right]\left[\begin{array}{cc} cos\theta & sin\theta \\ -sin\theta & cos\theta \end{array}\right]=\;Ae^{i\varphi_{2}}\left[\begin{array}{cc} cos2\theta & sin2\theta \\ sin2\theta & -cos2\theta \end{array}\right],$$

From Equation (2), it can be concluded that the transmission matrix can be changed by changing the long axis or short axis of the structure and the angle of the rotating unit structure, thereby affecting the output light.

A linearly polarized plane wave can be decomposed into two circularly polarized lights. Considering the two circularly polarized lights as: $E_{in}^{L}=\frac{\sqrt{2}}{2}E_{L}e^{i\delta_{L}}\left[\begin{array}{c} 1\\ i \end{array}\right]$ and $E_{in}^{R}=\frac{\sqrt{2}}{2}E_{R}e^{i\delta_{R}}\left[\begin{array}{c} 1\\ -i \end{array}\right]$, respectively, at this time, the reflected output light of the structure is:

(3) $$E_{out}^{LL}=\frac{\sqrt{2}}{2}AE_{L}e^{i\delta_{L}}e^{i\varphi_{2}}e^{-2\theta }\left[\begin{array}{c} 1\\ i \end{array}\right]=\frac{\sqrt{2}}{2}AE_{L}e^{i\delta_{L}}e^{i(\varphi_{2}-2\theta )}\left[\begin{array}{c} 1\\ i \end{array}\right],$$

(4) $$E_{out}^{RR}=\frac{\sqrt{2}}{2}AE_{R}e^{i\delta_{R}}e^{i\varphi_{2}}e^{2\theta }\left[\begin{array}{c} 1\\ -i \end{array}\right]=\frac{\sqrt{2}}{2}AE_{R}e^{i\delta_{R}}e^{i\left(\varphi_{2}+2\theta \right)}\left[\begin{array}{c} 1\\ -i \end{array}\right],$$

Among them, $E_{out}^{LL}$ represents the left-handed component of the reflected output light under the incidence of left-hand circularly polarized light, and $E_{out}^{RR}$ represents the right-handed component of the reflected output light under the incidence of the right-handed circularly polarized light. ±2θ is the geometric phase.

For the radial/azimuthal vector light field with the same concentric polarization rotation direction, the interface phase distribution should satisfy [63]:

(5) $$\Phi \left(r,\alpha \right)=-2\pi r\frac{NA}{\lambda }+l\alpha ,$$

In Equation (5), *NA* is the numerical aperture, *NA* = *λ*/*P*, *λ* is the operating wavelength, *P* is the total period of the 2*π* phase along the radial direction, and l is the topological charge number. Therefore, the phase that needs to be designed can be expressed as: $\Phi_{LL}=\varphi_{2}-2\theta =-2\pi r/P-\alpha$, $\Phi_{RR}=\varphi_{2}+2\theta =-2\pi r/P+\alpha$. According to Equations (3) and (4), we can obtain:

(6) $$E_{out}^{LL}=\frac{\sqrt{2}}{2}AE_{L}e^{i\delta_{L}}e^{i(\varphi_{2}-2\theta )}\left[\begin{array}{c} 1\\ i \end{array}\right]=\frac{\sqrt{2}}{2}AE_{L}e^{i\delta_{L}}e^{i\left(-\frac{2\pi r}{P}-\alpha \right)}\left[\begin{array}{c} 1\\ i \end{array}\right],$$

(7) $$E_{out}^{RR}=\frac{\sqrt{2}}{2}AE_{R}e^{i\delta_{R}}e^{i\left(\varphi_{2}+2\theta \right)}\left[\begin{array}{c} 1\\ -i \end{array}\right]=\frac{\sqrt{2}}{2}AE_{L}e^{i\delta_{L}}e^{i\left(-\frac{2\pi r}{P}+\alpha \right)}\left[\begin{array}{c} 1\\ -i \end{array}\right],$$

It can be seen from Equation (6) that when the incident light is left-handed circularly polarized light, the left-handed component of the reflected light is a vortex beam with a topological charge of −1. Similarly, it can be observed from Equation (7) that when the incident light is right-handed circularly polarized light, the right-handed component of the reflected light is a vortex beam with a topological charge of +1. When a linearly polarized light is incident, the output light can be expressed as:

(8) $$E_{out}=\frac{\sqrt{2}}{2}E_{out}^{LL}+\frac{\sqrt{2}}{2}E_{out}^{RR}=AEe^{-i\frac{2\pi r}{P}}\left[\begin{array}{c} cos\left(\alpha +\beta \right)\\ sin\left(\alpha +\beta \right) \end{array}\right],$$

In Formula (8), when the linearly polarized light is incident along the x-direction (β = 0), the output light is radial vector light, and when the linearly polarized light is incident in the y-direction (β = π/2), the outgoing light is azimuthal vector light.

## 3. Design of Multi-Function Metasurfaces

**Figure 2:**
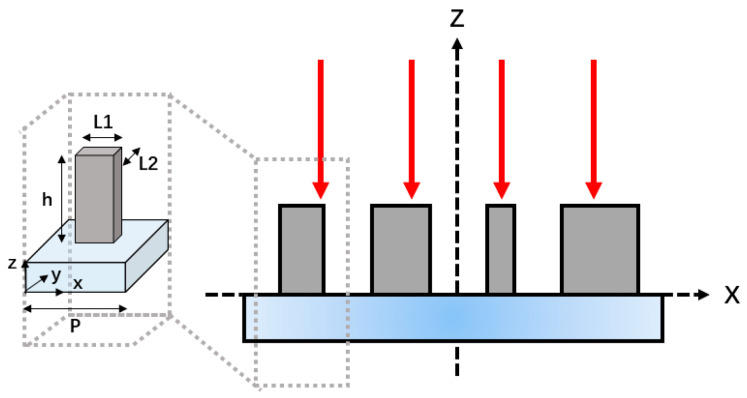
Schematic diagram of the reflective metasurface structure.

Figure 2 is a schematic diagram of a reflective metasurface, which is high-resistance silicon with a relative dielectric constant of 11.7 and a thickness of h = 180 μm on fused silica. The period P of the high resistance silicon pillar is 300 μm. By changing the lengths $L_{1}$ and $L_{2}$ in two orthogonal directions of the silicon pillar, its phase can be controlled to cover the entire 2π range [64]. At the same time, an additional PB phase is generated by rotating the silicon pillar. The metasurface acts as a half-wave plate, and a phase difference of π is required between the principal axes of the designed metasurface units. To improve the polarization conversion efficiency, we can consider the special cases of $A_{x}\approx A_{y}\approx A$ and $\varphi_{x}-\varphi_{y}=\pi$ in which the silicon pillar acts as a half-wave plate. In Equation (2), $\varphi_{2}$ is the phase of the silicon pillar along the $L_{2}$ direction, θ is the rotation angle of the structure, and 2θ is the geometric phase. Among them, the phase of the output light is changed by changing the length and width of the silicon pillar to meet the half-wave plate function, and the angle of the silicon pillar is rotated to meet different incident light polarization states, through which each metasurface unit is individually designed to achieve a specified polarization conversion.

**Figure 3:**
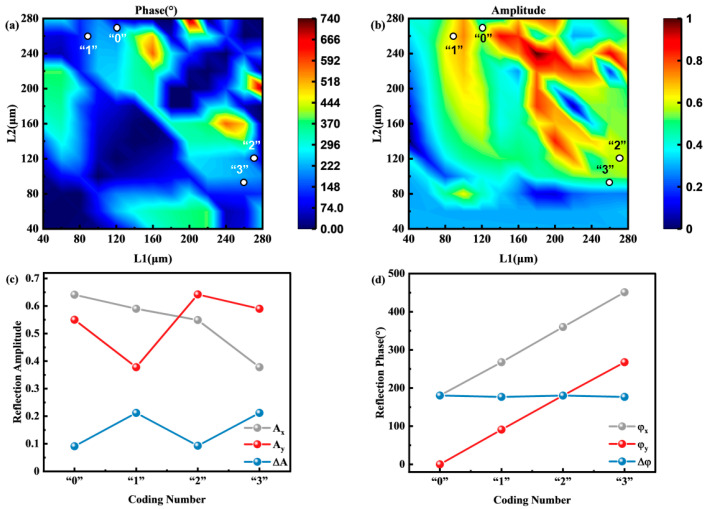
(**a**,**b**) The phase and amplitude spectra of silicon pillars changing $L_{1}$ and $L_{2}$ at 0.7 THz; the numbers in the figure indicate the size selection of the four coding structures. (**c**) The amplitude values $A_{x}$ along with the x-direction corresponding to the four coding structures; the amplitude value $A_{y}$ along the y-direction, and the amplitude difference ΔA. (**d**) The phase value $\varphi_{x}$ along with the x-direction corresponding to the four coding structures; the phase value $\varphi_{y}$ along the y-direction and the phase difference value Δφ.

Figure 3a,b show the phase and amplitude results of changing $L_{1}$ and $L_{2}$ of the silicon pillar at 0.7 THz. Accordingly, the corresponding operating wavelength is 428.6 μm. The full 2π phase regulation at 0.7 THz is achieved by changing the lengths of $L_{1}$ and $L_{2}$. The location of the selected four coding structures is marked in the figure. As shown in Table 1, the structure sizes of the four units are selected as $L_{1}$ = 120, 86, 270, 260 μm and $L_{2}$ = 270, 260, 120, 86 μm. Figure 3c shows the amplitude values $A_{x}$ under the incident polarization along the x-direction and the amplitude values $A_{y}$ under the incident polarization along the y-direction. The amplitude difference is $\Delta A=\left|A_{x}-A_{y}\right|$, corresponding to the four coding structures. Figure 3d shows the phase value $\varphi_{x}$ under the incident polarization along the x-direction and the phase value $\varphi_{y}$ under the incident polarization along the y-direction. The phase difference value is $\Delta \varphi =\varphi_{x}-\varphi_{y}$, corresponding to the four coding structures. The phase of four coding structures are linearly increased in 90° steps. At the same time, to ensure that all selected coding structures play the role of half-wave plates at the selected frequency of 0.7 THz, the phase differences Δφ are close to π. Based on these four coded silicon pillars, a metasurface was designed for generating vector beams.

**Figure 4:**
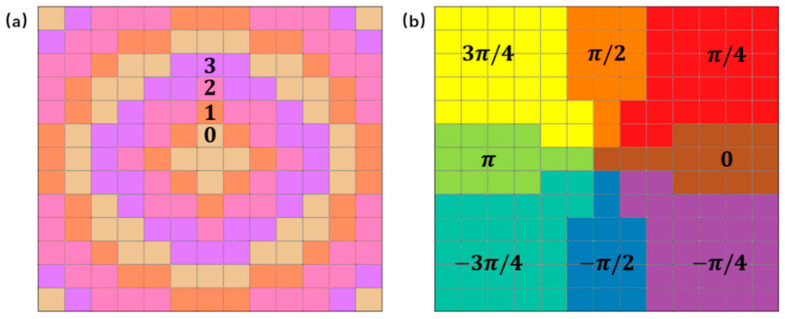
(**a**,**b**) Colors indicating the phase change of the two encoding methods.

Under the incident polarization along the x-direction, the emergent light is a radial vector beam, and under the incident polarization along the y-direction, the emergent light is an angular vector beam. To realize the above design, the four coding structures are firstly arranged according to radial diffusion. Different from this phase modulation, the metasurface is also evenly divided into eight regions, and the arranged coding structures in each region are rotated by the same angle *θ*. The *θ* angle is determined by the required phase α = 2θ, as shown in Figure 4. The phase changes of the two encoding methods are simply illustrated in Figure 4.

Among them, the four colors in Figure 4a represent four coding structures, respectively, that are determined by changing $L_{1}$ and $L_{2}$ to form the dynamic phase. The eight region phases α of Figure 4b are arranged in rotation from 0 to 2π, thereby forming the second PB phase. The designed metasurfaces are arranged according to the above arrangement.

**Figure 5:**
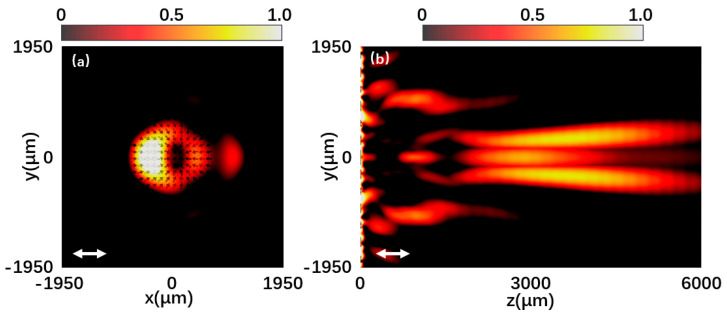
(**a**,**b**) Normalized intensity distribution at 0.7 THz under the x-polarized light incidence.

**Figure 6:**
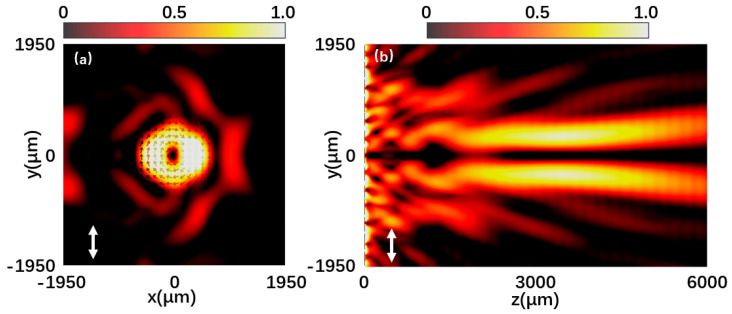
(**a**,**b**) Normalized intensity distribution at 0.7 THz under the y-polarized light incidence.

Silicon dioxide can be deposited on high-resistance silicon wafers by plasma chemical vapor deposition. The silicon wafer was directly bonded to the fused silica wafer by spin-on adhesive followed by UV exposure. The photoresist lithography and deep reactive ion etching process are etched using traditional mask lithography technology. High-resistance silicon is widely used in terahertz optics due to its low loss and low dispersion in the terahertz spectrum. The metasurface is composed of 13 × 13-unit structures. Figure 5a is the normalized intensity distribution at 0.7 THz under the incidence of x-polarized light. It can be seen that the light intensity distribution is a doughnut shape with a hollow center. The intensity outside this shape is relatively low, and the arrows in the figure indicate the polarization distribution, which is radially directed toward the concentricity. The polarization states represented by the arrows are the calculated results of the vector electric field obtained by the simulation. Figure 5b shows the yz plane intensity distribution of the generated vector beam under the incident x-polarized light without diffraction characteristics. Figure 6a shows the normalized intensity distribution at 0.7 THz under the incidence of y-polarized light. The normalization factor is $\left({\left|E\right|}^{2}-{\left|E\right|}_{min}^{2})/({\left|E\right|}_{max}^{2}-{\left|E\right|}_{min}^{2}\right)$, and ${\left|E\right|}^{2}$ represents the intensity of the plane electric field. It can also be clearly seen that the light intensity distribution is in the shape of a donut with a hollow center. The polarization distribution of the arrow in the figure is an angular distribution with the same rotation direction. Figure 6b is the intensity distribution of the yz plane under the incident y-polarized light. It can be seen that the emergent beam has a long non-diffraction distance of about 10λ. The results are very consistent with the idea of the theoretical design. Under the incidence of y-polarized light, the halo of azimuthal vector beams generated by the metasurface is more uniform. However, under the incidence of x-polarized light, the radial vector beam halo generated by the metasurface has poor uniformity. Fortunately, by increasing the number of coding metaunits to refine the phase change, this inhomogeneity can be reduced. It can also be optimized by increasing the resolution in the pattern design. When designing the units, the amplitude and phase of each cell should be relatively uniform.

**Figure 7:**
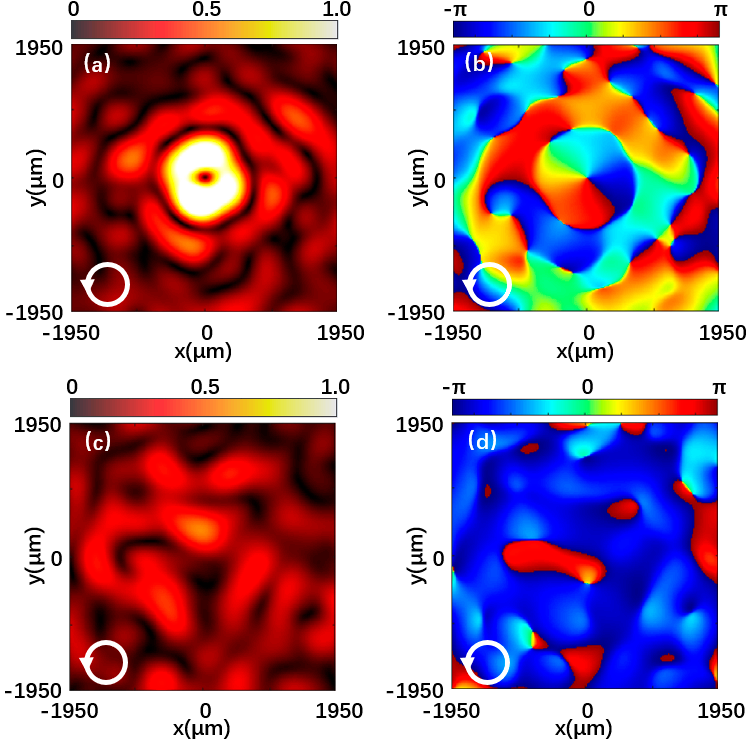
Reflected beam at 0.7 THz under the left-handed polarized light incidence. (**a**,**b**) Normalized intensity distribution and phase distribution of the left-handed component. (**c**,**d**) Normalized intensity distribution and phase distribution of the right-handed component.

**Figure 8:**
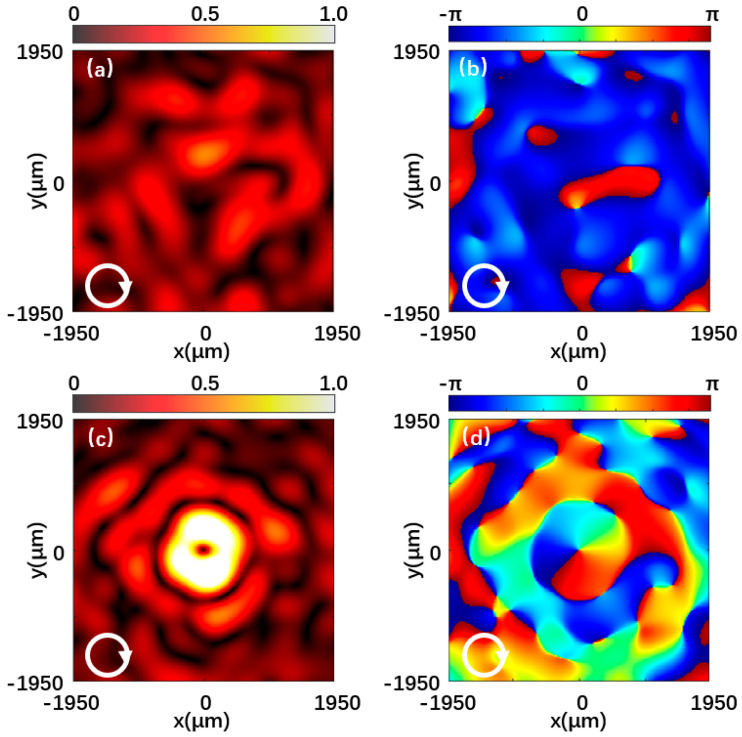
Reflected beam at 0.7 THz under the right-handed polarized light incidence. (**a**,**b**) Normalized intensity distribution and phase distribution of the left-handed component. (**c**,**d**) Normalized intensity distribution and phase distribution of the right-handed component.

Figure 7 and Figure 8 are the left-handed and right-handed component intensities and phase distributions when the metasurface is illuminated by left-handed circularly polarized light and right-handed circularly polarized light, respectively. Figure 7a is the normalized intensity distribution of the left-handed component of the reflected beam at 0.7 THz under left-handed polarized light incidence. A hollow doughnut-shaped vortex beam in the center is shown. Figure 7b shows the phase distribution corresponding to the left-handed component, and the helical phase distribution indicates that the output light is a vortex beam with a topological charge of −1. In contrast, the intensity distribution and phase distribution of the right-handed component in Figure 7c,d are irregular. Likewise, in Figure 8, the left-handed and right-handed components exhibit the same theoretical effect when the incident light polarization is opposite. The simulation results provide a good demonstration of the vortex beam properties of the generated beam.

The OAM patterns were quantitatively analyzed by employing Fourier transform analysis. The formula is as follows [65]:

(9) $$A_{l}=\frac{1}{2\pi }\int_{0}^{2\pi }\psi \left(\varphi \right)e^{-jl\varphi }d\varphi ,$$

(10) $$\psi \left(\varphi \right)=\sum_{l}A_{l}e^{jl\varphi },$$

**Figure 9:**
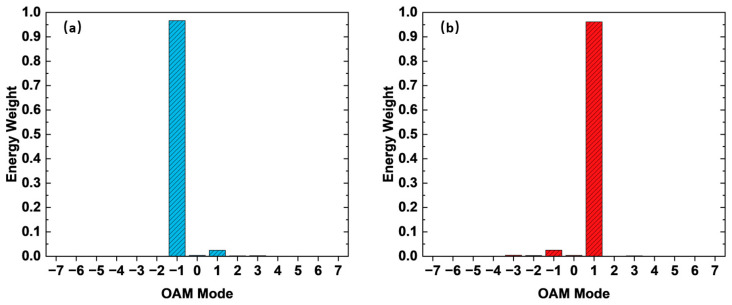
OAM spectrum. (**a**) Topological charge distribution of the left-handed component under the incidence of left-handed polarized light. (**b**) Topological charge distribution of the right-handed component under the incidence of right-handed polarized light.

As shown in Figure 9, the OAM topological charge number l = −7 to 7 is selected, and the energy weight of the OAM topological charge number is defined as:

(11) $$\mathrm{energy}\;\mathrm{weight}=\frac{{A_{l}}^{2}}{\sum_{l^{\prime }=-7}^{7}{A_{l^{\prime }}}^{2}},$$

It can be seen that for the left-handed component in Figure 9a, the topological charge number l=−1 accounts for the main part of the component, and the remaining topological charge number components are relatively small. In the right-handed component in Figure 9b, the topological charge number l=1 accounts for the main component.

## 4. Conclusions

We propose a method for generating vector beams in the terahertz band using an all-dielectric reflection-type metasurface. By using the superposition of the dynamic phase and the PB phase of the encoding unit structure at the same time, the generation of the vector beams under the incidence of linearly polarized light can be realized. The metasurface is composed of four kinds of coded cell structures, all of which have a half-wave plate function and fully cover the 2π phase. The proposed reflective metasurface has potential applications in generating vector beams with high integration. The method can be extended to other frequency ranges.

## Figures and Tables

**Table 1:** Parameter settings. (Unit: μm).

Encoding Particles	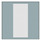	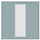	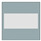	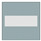
$L_{1}$/x	120	86	270	260
$L_{2}$/y	270	260	120	86
Digital coding number	“0”	“1”	“2”	“3”

## Data Availability

The data presented in this study are openly available.
